# Taxonomy-Guided Discovery of Antiviral Resources in Korean *Peucedanum* L. (Apiaceae)

**DOI:** 10.3390/ijms27198676

**Published:** 2026-09-28

**Authors:** Gun Lee, Kyeonghee Kim, Seung Mok Ryu, Heewon Ji, Hyun Ji Lee, Hyeon Jeong Moon, Chung-Do Lee, Jaewoo Choi, Yeong-Bin Baek, Sang-Ik Park, Joong-Sun Kim, Jun-Gyu Park, Jun-Ho Song

**Affiliations:** 1 Department of Veterinary Pathology, College of Veterinary Medicine and BK21 FOUR Program, Chonnam National University, Gwangju 61186, Republic of Korea; udlrjs77@naver.com (G.L.); dals404@naver.com (H.J.M.); cndehsla@gmail.com (C.-D.L.); e05311cht@naver.com (J.C.); ybbaek@jnu.ac.kr (Y.-B.B.); sipark@jnu.ac.kr (S.-I.P.); 2 Department of Veterinary Zoonotic Diseases, College of Veterinary Medicine, Chonnam National University, Gwangju 61186, Republic of Korea; 3 Herbal Medicine Resources Research Center, Korea Institute of Oriental Medicine, Naju 58245, Republic of Korea; kimk1228@kiom.re.kr (K.K.); smryu@kiom.re.kr (S.M.R.); hyunji@kiom.re.kr (H.J.L.); 4 Department of Biology, Chungbuk National University, Cheongju 28644, Republic of Korea; hw513513@naver.com; 5 Animal Medical Institute, Chonnam National University, Gwangju 61186, Republic of Korea; centraline@jnu.ac.kr; 6 Department of Veterinary Anatomy, College of Veterinary Medicine and BK21 FOUR Program, Chonnam National University, Gwangju 61186, Republic of Korea; 7 Advanced-Basic-Convergence Research Institute, Chungbuk National University, Cheongju 28644, Republic of Korea

**Keywords:** *Peucedanum*, Apiaceae, influenza A virus, antiviral activity, taxonomy-guided bioprospecting, chemotaxonomy, natural products

## Abstract

The genus *Peucedanum* (Apiaceae) contains diverse coumarins and related secondary metabolites with a wide range of pharmacological activities; however, the antiviral potential of Korean taxa remains largely unexplored. In this study, we evaluated the cytotoxicity and anti-influenza activity of aerial-part extracts from Korean *Peucedanum* taxa and the allied species *Sillaphyton podagraria*. Twenty-six ethanol extracts, comprising 24 accessions representing eight identified taxa and two unidentified *Peucedanum* samples, were screened against influenza A/Puerto Rico/8/34 (H1N1) virus in MDCK cells. Cytotoxicity and antiviral activity were assessed using MTT-based assays, and the half-maximal inhibitory concentration (IC_50_), half-maximal cytotoxic concentration (CC_50_), and selectivity index (SI) were determined. Most extracts showed low cytotoxicity, with CC_50_ values > 100 μg/mL. *P. terebinthaceum* var. *deltoideum* showed the strongest and most consistent activity across all three accessions, with IC_50_ values of 6.42 to 12.18 μg/mL and SI values ranging from >8.21 to >15.57. One *P. japonicum* extract (Pj2) was also active (IC_50_ = 15.52 μg/mL; SI > 6.44). Comparative chromatographic profiling further revealed taxon-specific chemical patterns. Collectively, these findings identify *P. terebinthaceum* var. *deltoideum* as a promising source of antiviral compounds and highlight the utility of a taxonomy-guided approach for bioprospecting and natural-product-based antiviral discovery.

## 1. Introduction

The family Apiaceae Lindl. (= Umbelliferae Juss.) is particularly notable for its diverse secondary metabolites, including coumarins, furanocoumarins, flavonoids, and essential oils, many of which exhibit antiviral, anti-inflammatory, and immunomodulatory activities [1,2]. Among its genera, *Peucedanum* L. has attracted considerable attention because of its medicinal importance and remarkable chemical diversity. Several *Peucedanum* species have long been used in traditional medicine and are known to contain abundant coumarins and other bioactive metabolites [3,4,5,6]. A representative example is *P. praeruptorum* Dunn (Peucedani Radix), a medicinal plant widely used in East Asia that is particularly rich in angular-type pyranocoumarins with reported anti-inflammatory, antitussive, and antiviral activities [7]. Similarly, *P. japonicum* Thunb. (Peucedanum Japonici Radix) contains a variety of coumarins, caffeoylquinic acids, flavonoids, and other phenolic compounds associated with antioxidant, anti-inflammatory, anticancer, and antiviral activities [5,8,9,10]. Collectively, these findings highlight *Peucedanum* as a promising source of bioactive secondary metabolites with potential applications in pharmaceutical research and drug discovery.

Traditionally circumscribed, *Peucedanum* sensu lato (s.l.) comprises approximately 120 species distributed across temperate regions of Europe and Asia [11]. Members of the genus are characterized by compound umbels, pinnately divided leaves, and schizocarpic fruits consisting of two mericarps with winged marginal ribs and well-developed vittae containing aromatic oils [12,13,14]. Recent taxonomic and phylogenetic studies, however, have demonstrated that *Peucedanum* s.l. is highly heterogeneous and represents a complex assemblage of morphologically similar but evolutionarily distinct lineages [15,16,17,18,19]. Molecular phylogenetic analyses have led to the recognition of several segregate genera and a reassessment of species boundaries within the group [11].

In Africa, species of *Peucedanum* s.l. have been redistributed among six distinct genera, three of which were newly established in that study, highlighting the substantial phylogenetic divergence among these lineages and supporting their recognition as separate genera [16]. With respect to Chinese species of *Peucedanum* s.l., molecular phylogenetic analyses have resulted in the transfer of 13 species to the genera *Sinopeucedanum* B.N. Song, C.K. Liu & X.J. He and *Shanopeucedanum* B.N. Song, C.K. Liu & X.J. He [20]. In Northeast Asia, Pimenov established the genus *Kitagawia* Pimenov in 1986 based on morphological evidence and included 10 species in the genus [21,22]. Recent phylogenetic analyses of Korean species traditionally assigned to *Peucedanum* s.l. indicated that all species except *P. japonicum* [≡ *Si. japonicum* (Thunb.) C.K. Liu & X.J. He] should be placed in *Kitagawia* [19]. Such taxonomic revisions raise important questions as to whether evolutionary divergence among lineages is accompanied by corresponding differences in phytochemical composition and biological activities. Increasing evidence from medicinal plant research suggests that taxonomic relationships can help predict secondary metabolite diversity and pharmacological potential, thereby providing the basis for taxonomy-guided bioprospecting strategies.

The continued emergence and re-emergence of viral infectious diseases represents a global public health concern, with influenza viruses among the most significant respiratory pathogens affecting both humans and animals and causing seasonal epidemics as well as occasional pandemics with substantial socioeconomic impacts [23]. Although approximately 100 antiviral drugs are currently available, their effectiveness is often limited by narrow therapeutic windows and the rapid emergence of resistant viral strains [24]. Resistance-associated mutations have been reported for all major classes of anti-influenza drugs, including M2 ion channel inhibitors, neuraminidase inhibitors, and polymerase inhibitors, highlighting the urgent need for novel antiviral agents with alternative mechanisms of action [25,26].

Natural products have historically served as a rich source of therapeutic compounds, and medicinal plants continue to provide structurally diverse metabolites with broad pharmacological activities [27]. More specifically, plant-derived secondary metabolites may exhibit multi-target modes of action, which may offer advantages in addressing the development of antiviral resistance [28]. Numerous medicinal plants have demonstrated antiviral activity against influenza viruses through various mechanisms, including the inhibition of viral entry, replication, and release, as well as the modulation of host inflammatory responses [29,30]. These findings highlight the potential of medicinal plants as valuable sources of novel antiviral compounds.

The Korean Peninsula harbors considerable diversity of *Peucedanum* and related taxa. Currently, seven taxa of *Peucedanum* and one closely related species, *Sillaphyton podagraria* (H. Boissieu) Pimenov (= *P. insolens* Kitag.), are recognized in Korea, occupying diverse habitats ranging from coastal cliffs and forest margins to mountainous regions [31,32]. These taxa exhibit substantial variation in vegetative and reproductive morphology, which may also be associated with differences in their chemical and biological characteristics. Despite their taxonomic diversity and potential medicinal value, comparative studies evaluating antiviral activity across Korean *Peucedanum* s.l. taxa remain scarce. To date, most pharmacological investigations have focused primarily on *P. japonicum*, while other Korean taxa remain largely unexplored.

Therefore, the present study investigated eight taxa representative of Korean *Peucedanum* s.l. and its allied lineage, *Sillaphyton*. By integrating taxonomic sampling with antiviral screening, we aimed to (1) evaluate the cytotoxic and anti-influenza activities of aerial-part extracts from representative Korean taxa, (2) compare biological activities among taxonomically distinct lineages, and (3) assess the potential of Korean *Peucedanum* s.l. and related taxa as sources of antiviral natural products. Through this approach, we further sought to evaluate the utility of taxonomic diversity as a framework for taxonomy-guided bioprospecting and the identification of promising antiviral plant resources.

## 2. Results

### 2.1. Morphological Diversity Among Korean Peucedanum s.l. Taxa

The examined taxa exhibited substantial morphological variation in vegetative and reproductive characters (Figure 1; Table 1). Differences were particularly evident in leaf architecture, ultimate leaflet morphology, umbel structure, and fruit shape. The taxa could be broadly separated into two groups: (1) taxa with ternately divided leaves (*P. japonicum*, *P. hakuunense* Nakai, and *Sillaphyton podagraria*) and (2) taxa with pinnately divided leaves (*P. chujaense* K. Kim, S.H. Oh, Chan S. Kim & C.W. Park, *P. terebinthaceum* (Fisch. ex Trevir.) Turcz. var. *terebinthaceum*, *P. terebinthaceum* var. *deltoideum* Makino ex Y. Yabe, *P. miroense* K. Kim, H.J. Suh & J.H. Song, and *P. tongkangense* K. Kim, H.J. Suh & J.H. Song). Considerable variation was also observed in the number of rays per compound umbel, the number of flowers per umbel, and mericarp morphology (Table 1 and Table 2).

Among the investigated taxa, *P. terebinthaceum* var. *deltoideum* was distinguished by its ovate to oblong ultimate leaf segments, whereas *P. terebinthaceum* var. *terebinthaceum* had narrower oblong to lanceolate segments (Table 2). *P. japonicum* was characterized by glaucous leaf surfaces and relatively robust stems, while *S. podagraria* exhibited the smallest stem diameter and had ovoid to ellipsoid mericarps (Table 2). These morphological differences highlight the morphological diversity among Korean members of *Peucedanum* s.l. taxa and allied taxa.

### 2.2. Cytotoxicity of Peucedanum and Sillaphyton Extracts

The cytotoxicity of 26 extracts derived from *Peucedanum* s.l. and *Sillaphyton* taxa was evaluated in MDCK cells using an MTT assay. None of the three *Sillaphyton* extracts exhibited cytotoxicity, while 20 of the 23 *Peucedanum* extracts showed no cytotoxic effects, with CC_50_ values exceeding 100 µg/mL (Table 3). Only three extracts (Ph3, Pt3, and Pm2) exhibited CC_50_ values below 100 µg/mL, ranging from 81.706 to 94.812 µg/mL (Table 3). These results indicate that most of the tested extracts exhibited low cytotoxicity in MDCK cells within the tested concentration range.

### 2.3. Anti-Influenza Activity of Korean Peucedanum and Sillaphyton Taxa

The antiviral activities of the 26 extracts against influenza A/Puerto Rico/8/34 (PR8, H1N1) virus were evaluated in MDCK cells. To conservatively prioritize extracts with meaningful antiviral selectivity and potential for further investigation, extracts with an IC_50_ < 100 µg/mL and an SI > 5 were classified as active. This SI threshold was intentionally set higher than the SI of the positive control, chloroquine (IC_50_ = 11.262 µg/mL; SI = 2.220). Based on these criteria, four extracts (Pd1, Pd2, Pd3, and Pj2) were classified as active (Figure 2; Table 3). Among them, the three *P. terebinthaceum* var. *deltoideum* extracts (Pd1, Pd2, and Pd3) exhibited the strongest and most consistent antiviral activity (Table 3). Pd1 showed the lowest IC_50_ value (6.424 µg/mL) and the highest SI (>15.566), followed by Pd2 (IC_50_ = 9.636 µg/mL; SI > 10.377) and Pd3 (IC_50_ = 12.18 µg/mL; SI > 8.210). Pj2 was also classified as active, with an IC_50_ of 15.52 µg/mL and an SI of >6.443 (Table 3). Pb2, Pj1, Sd1, and Sd2 showed measurable inhibitory effects, but their minimum SI values did not meet the predefined threshold of 5 (Table 3). The remaining extracts failed to meet one or both predefined activity criteria (Table 3).

### 2.4. Chemical Profiling and Annotation of Characteristic Constituents in Peucedanum terebinthaceum Var. deltoideum

Among the investigated *Peucedanum* taxa, *P. terebinthaceum* var. *deltoideum* (Pd) extracts exhibited the strongest antiviral activity (Table 3). To characterize their chemical composition and identify representative constituents contributing to the observed metabolite profiles, the extracts were analyzed using UHPLC-QTOF-MS.

Representative UHPLC-PDA chromatograms (254 nm) of three independent Pd extracts (Pd1–Pd3) exhibited highly comparable chromatographic patterns, indicating a high degree of consistency in their major chemical profiles (Figure 3). Seven major peaks were consistently detected in all three extracts and were subsequently annotated using UHPLC-QTOF-MS in conjunction with an in-house Personal Compound Database and Library (PCDL).

Compound annotation was performed by comparing accurate mass, molecular formula, retention time, and characteristic adduct ions with an in-house PCDL constructed using authentic reference standards. Based on these comparisons, the seven characteristic constituents were identified as apiosylskimmin (1), chlorogenic acid (2), eleutheroside B (3), apterin (4), kaempferol 3-robinobioside (5), nicotiflorin (6), and decursin (7) (Table 4). The identified constituents comprised glycosylated coumarins (apiosylskimmin and apterin), flavonoid glycosides (kaempferol 3-robinobioside and nicotiflorin), a caffeoylquinic acid (chlorogenic acid), a phenylpropanoid glycoside (eleutheroside B), and the pyranocoumarin decursin, collectively constituting the characteristic chemical profile of the Pd extracts.

## 3. Discussion

The present study revealed that antiviral activity against influenza A virus was not uniformly distributed among Korean members of *Peucedanum* s.l. and allied taxa. Instead, meaningful antiviral activity was concentrated in a limited number of extracts, particularly those from all three accessions of *P. terebinthaceum* var. *deltoideum* and one accession of *P. japonicum* (Pj2). In contrast, none of the extracts from *P. chujaense*, *P. hakuunense*, *P. terebinthaceum* var. *terebinthaceum*, *P. tongkangense*, *P. miroense*, and *S. podagraria* met the predefined activity criterion (SI > 5) under the experimental conditions.

This pattern suggests that antiviral potential may be associated with taxonomic identity rather than representing a generalized characteristic of the genus *Peucedanum* s.l. Notably, extracts from all three accessions of *P. terebinthaceum* var. *deltoideum* consistently exhibited strong antiviral activity despite being collected from different populations. In contrast, only one of the three *P. japonicum* accessions (Pj2) met the predefined activity criterion, whereas none of the *S. podagraria* accessions met this threshold. The reproducible activity across geographically distinct accessions suggests that *P. terebinthaceum* var. *deltoideum* may represent a particularly promising taxon for further investigation of its antiviral potential.

Among the active extracts, Pd1 exhibited the most potent antiviral activity (IC_50_ = 6.424 µg/mL, SI > 15.57), with a lower IC_50_ and higher SI than the positive control chloroquine (IC_50_ = 11.26 µg/mL, SI = 2.220) under the present experimental conditions. To place these findings in the context of previously reported plant-derived anti-influenza agents, representative studies were compared with the present results. Ginsenoside rk1 from *Panax ginseng* C.A. Mey. showed activity against influenza A/PR8 (H1N1), with a reported EC_50_ = 6.2–14.8 µM [33]. Another plant-derived extract, a butanol fraction of *Davallia mariesii* H.J. Veitch, exhibited anti-influenza activity (EC_50_ = 24.32 µg/mL, SI = 6.05) [34]. Similarly, a 40% ethanolic leaf extract of *Spiraea salicifolia* L. exhibited pronounced activity against influenza A (H1N1), with an IC_50_ of 5.9 µg/mL and an SI of 10 [35], placing its activity within a range comparable to that observed for the Pd extracts in the present study. Although these findings provide useful context for the activity observed in the Pd extracts, direct quantitative comparisons should be interpreted cautiously because of differences in extract preparation or compound purity, viral strains, host cell lines, assay procedures, and concentration units. The two additional *P. terebinthaceum* var. *deltoideum* extracts (Pd2: IC_50_ = 9.636 µg/mL, SI > 10.377; Pd3: IC_50_ = 12.186 µg/mL, SI > 8.210) showed similarly strong activity, further supporting the potential of this variety as a promising source of anti-influenza compounds. Although Pb2 showed measurable inhibitory effects, it did not meet the predefined activity criterion (SI > 5). Among the three *P. japonicum* extracts, only Pj2 met the predefined activity criterion (IC_50_ = 15.520 µg/mL, SI > 6.443). Pj1 showed measurable inhibitory effects (IC_50_ = 29.911 µg/mL, SI > 3.343) but did not meet the SI > 5 criterion, while Pj3 was inactive (IC_50_ > 100 µg/mL). These results are consistent with previous reports of the inhibitory effects of *P. japonicum* constituents on influenza virus replication [5] and extend those findings by providing the quantitative IC_50_ and SI data against the PR8 strain. *P. japonicum* is widely used as a medicinal plant, particularly in East Asia [36,37]. According to the Korean Pharmacopoeia (2024), the crude drug derived from this species is listed as ‘Peucedani Japonici Radix’, and its local name is recorded as ‘Sig-Bang-Poong’. In Japan, it has also been used in folk medicine for centuries under the names ‘Chomeiso’ and ‘Botanbofu’ [36,38]. In recent years, various cosmetic products and functional foods incorporating extracts of this plant have been developed [38].

The inter-accession variation observed among *P. japonicum* extracts, with one extract meeting the predefined activity criterion and the other two showing lower or no detectable activity, may reflect differences in collection site, phenological stage, or post-harvest processing, all of which are known to influence secondary metabolite composition. Regional variation in *P. japonicum* phytochemical profiles has been documented previously [10], supporting this interpretation. The lower selectivity observed for the *P. japonicum* extracts (SI > 3.343 for Pj1 and >6.443 for Pj2) compared with *P. terebinthaceum* var. *deltoideum* suggests that further bioassay-guided fractionation and characterization of the active constituents are warranted before their antiviral potential can be more fully evaluated.

*Sillaphyton podagraria* showed measurable inhibitory effects in two accessions (Sd1: IC_50_ = 20.130 µg/mL, SI > 4.967; Sd2: IC_50_ = 20.193 µg/mL, SI > 4.952); however, neither extract met the predefined activity criterion (SI > 5) (Table 3). The similar inhibitory effects observed across these accessions suggest that this activity may be associated with shared chemical constituents. *S. podagraria* has been reported to contain diverse coumarins and flavonoids that may underlie the observed inhibitory effects; however, dedicated phytochemical characterization of this species remains limited and warrants further investigation.

The occurrence of antiviral activity in particular taxa highlights the potential value of taxonomic sampling in natural product discovery. Rather than relying solely on random screening of medicinal plants, systematic evaluation based on taxonomic diversity may provide a useful framework for identifying biologically active lineages and their associated metabolites.

The antiviral activity observed in the active extracts may be associated with the diverse array of coumarins, furanocoumarins, flavonoids, and phenolic compounds known to occur within *Peucedanum*. Members of *Peucedanum* s.l. are particularly rich in coumarins, including pyranocoumarins and furanocoumarins, many of which have been reported to exhibit antiviral, anti-inflammatory, and immunomodulatory activities [4,38]. These and other bioactive secondary metabolites may contribute to the antiviral effects observed in the present study. The mechanisms by which these compounds may exert antiviral effects are likely multifaceted, potentially involving effects on multiple stages of the viral life cycle. Praeruptorin A, a major coumarin constituent reported in several *Peucedanum* species, has been shown to inhibit influenza A H1N1 virus by targeting neuraminidase activity [28], a key viral surface protein involved in the release and spread of progeny virions. This mechanism is consistent with the antiviral target of oseltamivir and other neuraminidase inhibitors, but natural coumarins may offer the advantage of reduced resistance development due to their structural diversity and potential for multi-target effects. These findings suggest that coumarin derivatives may represent useful candidates for further investigation as naturally derived inhibitors of influenza virus neuraminidase.

Furanocoumarins, including peucedanin, 8-methoxypeucedanin, and bergapten, have been reported in *Peucedanum* species [39] and exhibit diverse biological activities, including antiviral activity. These compounds have also been reported to induce apoptosis, inhibit cell cycle progression, and modulate inflammatory responses [39,40]. Although these biological activities may be relevant to their pharmacological effects, their specific contributions to anti-influenza activity remain to be established. The anti-inflammatory properties of *Peucedanum* coumarins, including reported modulation of the NF-κB signaling pathway [41], may also provide additional biological effects relevant to the host inflammatory response during viral infection; however, their contribution to the antiviral effects observed in the present study requires further investigation.

Flavonoids and phenolic compounds reported from *Peucedanum* species, such as 4-caffeoylquinic acid, rutin, hyperoside, and hesperidin [42], may contribute to antiviral activity through multiple mechanisms. Flavonoids have been shown to interfere with viral attachment and entry by binding to viral surface proteins; inhibit viral enzymes, including neuraminidase and RNA-dependent RNA polymerase; and modulate host immune responses [29]. The combined presence of diverse bioactive constituents in crude extracts may contribute to the observed antiviral activity; however, potential additive or synergistic interactions among these compounds remain to be experimentally determined. Notably, Pd1 exhibited a lower IC_50_ and higher SI than the positive control chloroquine under the present experimental conditions.

The higher selectivity index of Pd1 (SI > 15.566) compared with chloroquine (SI = 2.220) is particularly noteworthy in the context of the present in vitro screening. A high selectivity index indicates that the extract can achieve effective antiviral concentrations without causing significant cytotoxicity, a critical requirement for safe therapeutic use. The presence of multiple bioactive constituents in the extract may contribute to its overall biological activity; however, the contributions and potential interactions of individual compounds remain to be determined. The concept of multi-component, multi-pathway, multi-target therapy has been well established in traditional medicine systems and is increasingly recognized in modern pharmacology as a strategy to achieve superior therapeutic outcomes with reduced side effects [43]. Accordingly, further bioassay-guided fractionation and evaluation of individual constituents and their combinations will be necessary to determine the chemical basis of the antiviral activity and selectivity observed for Pd1.

While this study provides valuable initial evidence for the antiviral potential of *Peucedanum* s.l. and related taxa, several limitations should be considered. First, the study employed crude extracts rather than purified compounds, which limits the ability to identify specific bioactive constituents responsible for the observed antiviral effects. Future research should focus on bioassay-guided fractionation to isolate and characterize the active compounds from the most potent extracts. Second, the mechanistic basis of the observed antiviral activity remains to be fully elucidated. Although the literature suggests that *Peucedanum* coumarins can inhibit neuraminidase and interfere with viral replication [28], the specific mechanisms by which the tested extracts exert their effects against the PR8 influenza virus require further investigation. Third, the inter-accession variation observed in some taxa, particularly *P. japonicum*, highlights the need to evaluate the effects of geographic origin, developmental stage, and environmental and post-harvest factors on phytochemical composition and antiviral activity. Expanded sampling across populations, together with comparative chemical profiling and antiviral assays, will be important for determining the consistency and taxonomic specificity of the observed activities.

An important implication of this study is that taxonomic diversity may provide a useful framework for exploring pharmacological diversity. The uneven distribution of antiviral activity among taxa suggests that antiviral potential may be associated with particular taxonomic groups rather than being uniformly distributed across *Peucedanum* s.l. and related taxa. This pattern is consistent with a chemotaxonomic perspective, in which variation in secondary metabolite profiles can provide information relevant to taxonomic relationships [44]. Previous studies have demonstrated that coumarin composition varies considerably among species of *Peucedanum* and related genera [4,39]. Therefore, the taxa exhibiting strong antiviral activity in the present study may possess distinctive phytochemical profiles associated with their observed biological activity, a hypothesis that should be tested through targeted metabolomic profiling and comparative bioactivity analyses.

From a practical perspective, these findings support the potential utility of taxonomy-guided bioprospecting strategies. By integrating taxonomic knowledge with biological screening, researchers can systematically prioritize taxonomically distinct groups for the investigation of novel bioactive compounds. Such an approach may provide a more structured framework for natural product discovery while simultaneously highlighting the value of plant biodiversity as a source of potentially bioactive natural products.

The present study demonstrates that antiviral activity varies substantially among Korean *Peucedanum* s.l. and related taxa, with particularly strong activity observed in specific taxa, and provides evidence that taxonomic diversity may represent a valuable framework for identifying promising antiviral resources. The integration of taxonomy, phytochemistry, and biological screening therefore offers a systematic framework for natural product-based antiviral discovery. More broadly, plant systematics and phylogenetic knowledge may have predictive value in guiding the selection of taxa with promising biological or chemical properties, rather than serving solely as tools for species identification and classification. Applying such taxonomy- and phylogeny-informed approaches to the exploration of diverse natural products, together with interdisciplinary integration of systematics, phytochemistry, pharmacology, and related fields, may facilitate the more targeted discovery of novel bioactive resources and provide new opportunities for natural product research and drug discovery.

## 4. Materials and Methods

### 4.1. Plant Materials and Extraction

This study included 24 accessions representing eight taxa [seven taxa of *Peucedanum* s.l. (*P. chujaense* K.Kim, S.H.Oh, Chan S.Kim & C.W.Park; *P. hakuunense* Nakai; *P. japonicum* Thunb.; *P. miroense* K.Kim, H.J.Suh & J.H.Song; *P. tongkangense* K.Kim, H.J.Suh & J.H.Song; *P. terebinthaceum* (Fisch. ex Trevir.) Turcz. var. *terebinthaceum*; *P. terebinthaceum* var. *deltoideum* Makino ex Y.Yabe) and *Sillaphyton podagraria* (H.Boissieu) Pimenov] (Table 1; Figure 1). In addition, two *Peucedanum* samples that could not be identified to species (Pasa and Paer) were included in the antiviral screening. Fresh whole-plant samples were collected from their natural habitats and taxonomically identified by J.-H. Song based on morphological characteristics. Voucher specimens were deposited in the herbaria of CBU and KIOM [acronyms follow Index Herbariorum; https://sweetgum.nybg.org/science/ih/ (accessed on 1 September 2026)]. For convenience, each extract was assigned a unique identifier based on its taxonomic origin and accession number. Extract IDs consisted of a species abbreviation followed by an accession number (e.g., Pd1–Pd3, Pj1–Pj3, Pb1–Pb3, and Sd1–Sd3) (Table 1). These extract IDs were used throughout the study for data presentation and analysis. Dried aerial parts, including stems and leaves (100 g per sample), of *Peucedanum* species were pulverized and extracted according to the previously established procedure [44]. Briefly, the powdered plant material (100 g) was subjected to three successive 1-h microwave-assisted extractions with 1 L of 70% ethanol, corresponding to a raw material-to-solvent ratio of 1:10 (*w*/*v*) per extraction cycle. The combined extracts were concentrated in vacuo at 35 °C and subsequently lyophilized to obtain dried extracts. The extraction conditions were adopted from our previously established protocol for comparative phytochemical profiling of Korean *Peucedanum* taxa [44], in which all plant materials were processed under identical extraction conditions.

### 4.2. Cells and Viruses

Madin–Darby canine kidney epithelial cells (MDCK; ATCC CCL-34) were obtained from the American Type Culture Collection (ATCC, Manassas, VA, USA). Cells were maintained in Dulbecco’s Modified Eagle’s Medium (DMEM) supplemented with 10% fetal bovine serum (FBS), 100 U/mL penicillin, and 100 µg/mL streptomycin. A representative influenza A virus (IAV) strain, A/Puerto Rico/8/34 (H1N1) (A/PR8), obtained from ATCC, was used for antiviral screening. MDCK cells are widely used for influenza virus propagation due to their high susceptibility and permissiveness to viral infection. The virus was propagated in MDCK cells cultured in serum-free DMEM supplemented with 1 µg/mL N-tosyl-L-phenylalanine chloromethyl ketone (TPCK)-treated trypsin (Sigma-Aldrich, St. Louis, MO, USA). Viral titers were determined using a cell culture-based immunofluorescence assay.

### 4.3. Antiviral Screening

A confluent monolayer of MDCK cells was prepared in a 96-well plate at a density of 5.0 × 10^4^ cells/well. The extracts were initially dissolved in DMSO at 50 mg/mL and subsequently diluted 500-fold with culture medium to obtain the highest test concentration of 100 µg/mL. Two-fold serial dilutions were then prepared to obtain final extract concentrations ranging from 100 to 1.56 µg/mL (100, 50, 25, 12.5, 6.25, 3.13, and 1.56 µg/mL). The final DMSO concentration was 0.2% (*v*/*v*) at the highest extract concentration and decreased proportionally with serial dilution (≤0.2% *v*/*v*). Cell viability was measured using an MTT assay as a percentage in comparison with DMSO-treated cells using GraphPad Prism software v9.5.1 (GraphPad Software, San Diego, CA, USA) as described previously [45].

To evaluate antiviral activity, a screening assay was performed using a modified method as previously described [44]. MDCK cells were pretreated with serially diluted plant extracts (100, 50, 25, 12.5, 6.25, 3.13, and 1.56 µg/mL) for 1 h. Subsequently, A/PR8 (H1N1) at a multiplicity of infection (MOI) of 0.04 was added in the presence of the corresponding concentration of plant extracts. Viral adsorption was carried out at 37 °C for 1 h in a medium supplemented with 1% penicillin–streptomycin (P/S) and 1 µg/mL TPCK-treated trypsin. After removing the inoculum and washing with Dulbecco’s phosphate-buffered saline (DPBS), the extracts were reapplied, and cells were incubated for an additional 48 h. Cell viability was assessed using the MTT assay according to the manufacturer’s instructions [45]. To determine the median inhibitory concentration (IC_50)_ and median cytotoxic concentration (CC_50_), data were fitted to a nonlinear regression model using an [Inhibitor] vs. normalized response—Variable slope model in GraphPad Prism software v9.5.1 (GraphPad Software, San Diego, CA, USA). The selectivity index (SI) was calculated as the ratio of CC_50_ to IC_50_ (SI = CC_50_/IC_50_).

### 4.4. Chemical Profiling by UHPLC-Q-TOF-MS Analysis

Chemical profiling was performed using an Agilent 1290 Infinity II UHPLC system (Agilent Technologies, Santa Clara, CA, USA) coupled with an Agilent 6546 quadrupole time-of-flight mass spectrometer (QTOF-MS) equipped with an electrospray ionization (ESI) source. Chromatographic separation was achieved on an ACQUITY UPLC BEH C18 column (2.1 × 100 mm, 1.7 μm; Waters Corp., Milford, MA, USA). The column temperature was maintained at 35 °C, and the autosampler temperature was set at 10 °C. The injection volume was 2 μL, and the flow rate was 0.4 mL/min.

The mobile phase consisted of 0.1% formic acid in water (A) and 0.1% formic acid in acetonitrile (B). Gradient elution was performed as follows: 5% B (0–1.0 min), 5–100% B (1.0–20.0 min), maintained at 100% B (20.0–22.3 min), returned to 5% B at 22.4 min, and equilibrated at 5% B until 25.0 min. UV chromatograms were acquired over 200–400 nm, and chromatograms at 254 nm were used for qualitative analysis.

Mass spectrometric detection was performed using an ESI source operated in positive and negative ion mode. The mass spectra were acquired over an *m*/*z* range of 100–1700. The optimized source parameters were as follows: drying gas temperature, 325 °C; drying gas flow, 8 L/min; nebulizer pressure, 35 psi; sheath gas temperature, 350 °C; sheath gas flow, 11 L/min; capillary voltage, 3500 V; fragmentor voltage, 175 V; skimmer voltage, 65 V; and octopole RF peak, 750 V. Continuous mass correction was performed using reference ions to ensure high mass accuracy throughout the analysis. Data acquisition and processing were carried out using MassHunter Acquisition and MassHunter Qualitative Analysis software (version 10.0, Agilent Technologies).

Compound annotation was performed using MassHunter Personal Compound Database and Library (PCDL) Manager (version 8.0, Agilent Technologies). An in-house PCDL database was established by registering authentic reference standards analyzed under identical UHPLC-QTOF-MS conditions. The database included compound names, molecular formulas, accurate masses, retention times, and MS/MS fragmentation information. Metabolites were tentatively annotated by matching accurate mass, isotope pattern, retention time, and MS/MS fragmentation spectra against the in-house PCDL database. For compounds available as authentic reference standards, compound identities were confirmed by comparison of retention time, accurate mass, isotope pattern, and MS/MS fragmentation spectra with the corresponding entries in the in-house PCDL. Structural isomers sharing identical molecular formulas and exact masses were distinguished based on their retention times and MS/MS fragmentation patterns.

## 5. Conclusions

This study provides the first comparative assessment of cytotoxicity and anti-influenza activity across Korean *Peucedanum* taxa and the allied species *Sillaphyton podagraria*. While most extracts exhibited low cytotoxicity, antiviral activity was unevenly distributed among taxa. Among the examined species, *P. terebinthaceum* var. *deltoideum* showed the strongest antiviral activity and the highest selectivity indices, while one *P. japonicum* extract (Pj2) also met this criterion. In contrast, little or no antiviral activity was detected in *P. hakuunense*, *P. tongkangense*, *P. miroense*, and *P. chujaense*. These findings indicate that antiviral potential varies substantially among taxa and accessions rather than being uniformly distributed across *Peucedanum* s.l. and related taxa.

The observed taxon-specific distribution of antiviral activity, together with differences in chemical profiles among the examined taxa, suggests that taxonomic diversity may be associated with variation in bioactive secondary metabolites. Our results therefore highlight the potential of Korean *Peucedanum* s.l. and related taxa as sources of antiviral natural products and demonstrate the utility of taxonomy-guided bioprospecting as a systematic framework for natural product research. Future studies integrating comparative metabolomics, molecular phylogenetics, and bioassay-guided fractionation and isolation will help identify the active constituents responsible for antiviral activity and further clarify the relationship between taxonomic diversity and pharmacological potential.

More broadly, plant systematics and phylogenetic knowledge may have predictive value in guiding the selection of taxa with promising biological and chemical properties, extending their application beyond species identification and classification. Integrating taxonomy- and phylogeny-informed approaches with phytochemistry, pharmacology, and biological screening may provide a more targeted framework for exploring diverse natural products. Such interdisciplinary approaches may facilitate the discovery of novel bioactive resources and expand the contribution of plant systematics to natural product research and drug discovery.

## Figures and Tables

**Figure 1:**
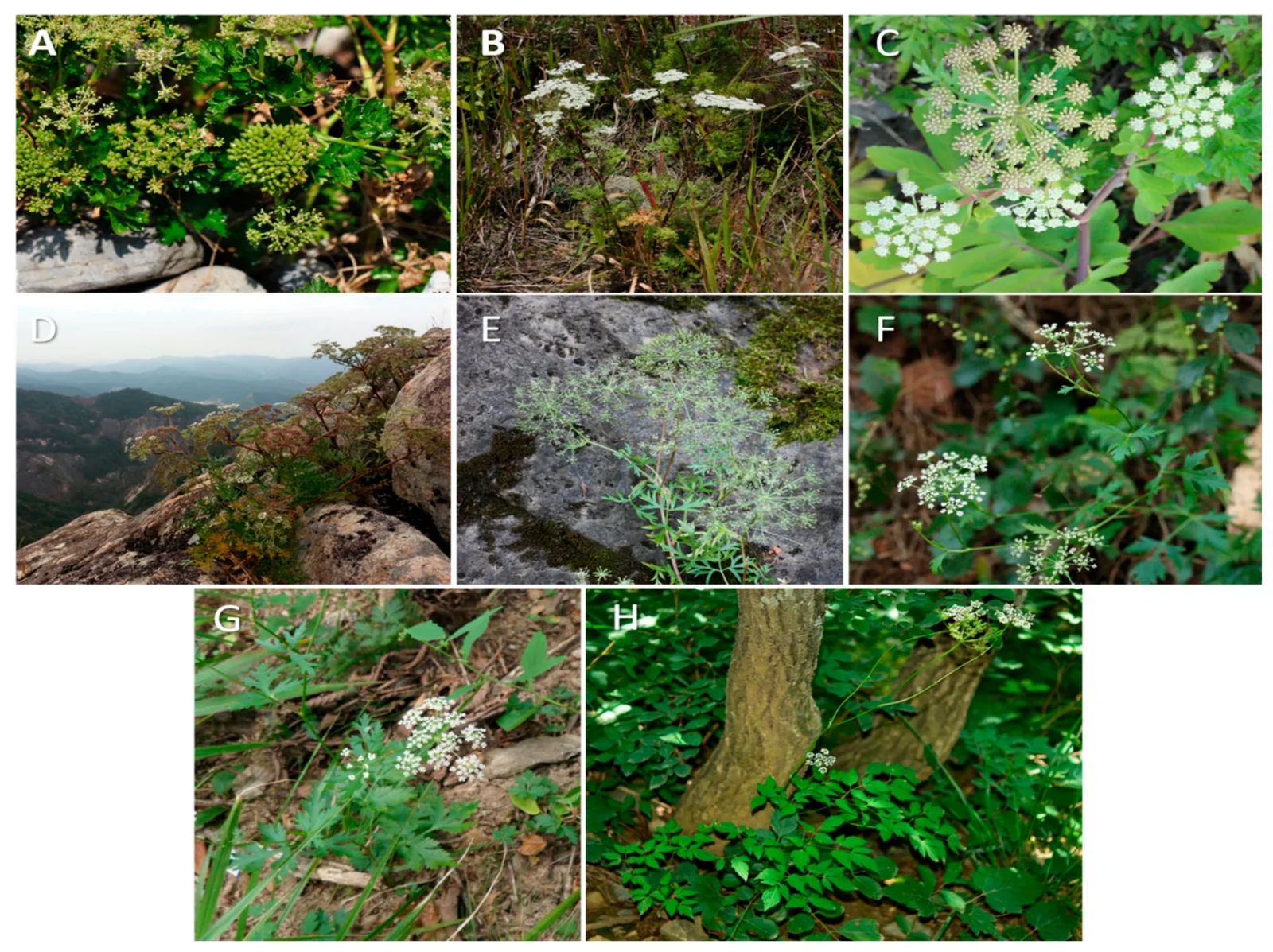
Photographs of Korean *Peucedanum* species: (**A**) *Peucedanum chujaense*; (**B**) *P. hakuunense*; (**C**) *P. japonicum*; (**D**) *P. miroense*; (**E**) *P. tongkangense*; (**F**) *P. terebinthaceum* var. *terebinthaceum*; (**G**) *P. terebinthaceum* var. *deltoideum*; (**H**) *Sillaphyton podagraria*.

**Figure 2:**
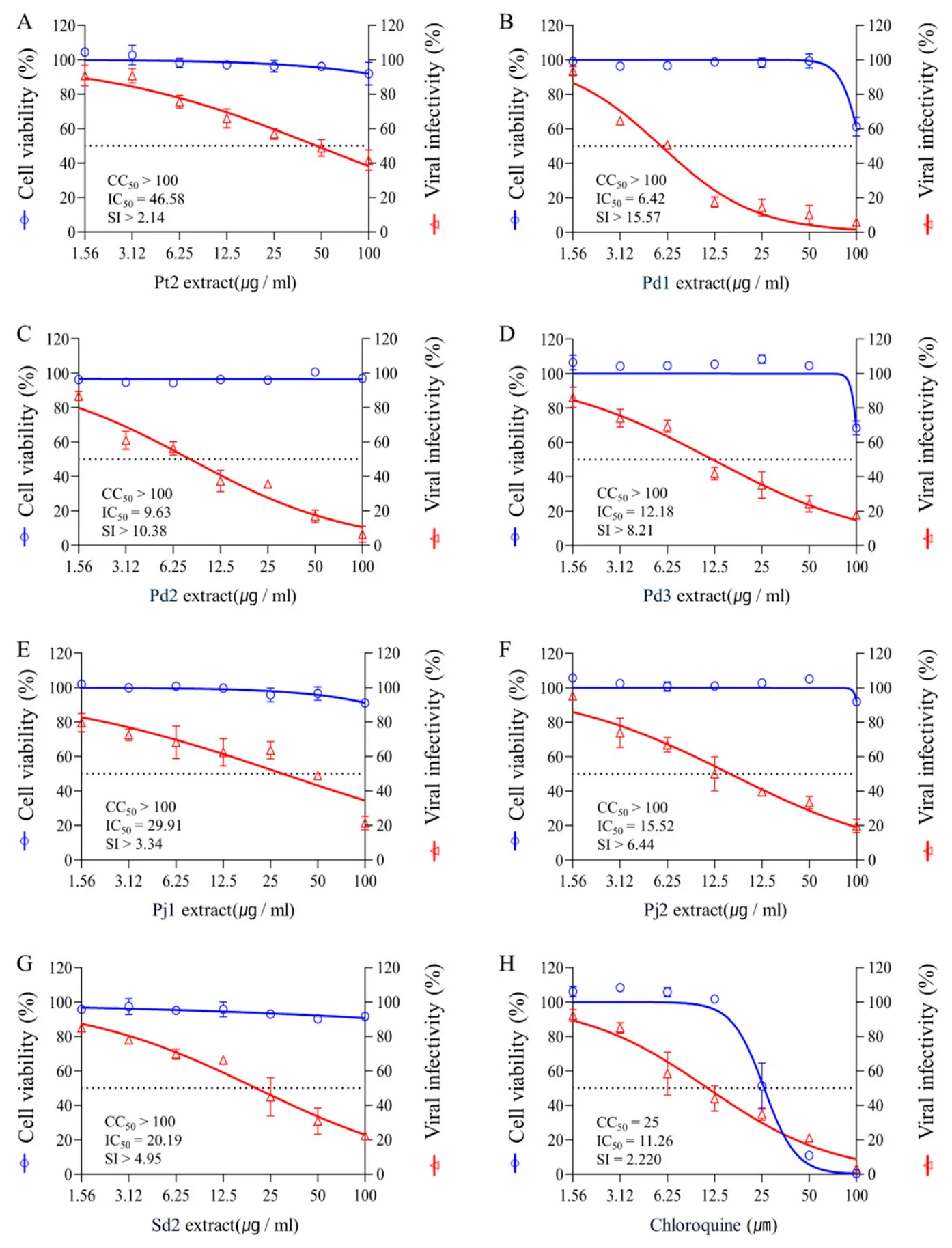
Antiviral screening of *Peucedanum* and *Sillaphyton* extracts against the influenza A/PR8 virus. MDCK cells were treated with serially diluted extracts (1.56, 3.13, 6.25, 12.5, 25, 50, and 100 µg/mL). Antiviral activity was assessed based on the inhibition of the cytopathic effect (CPE) induced by influenza A/PR8 virus in MDCK cells (MOI = 0.04). Cell viability (blue lines) and viral infectivity (red lines) were evaluated, and CC_50_ (µg/mL), IC_50_ (µg/mL), and SI values were calculated using GraphPad Prism 9.5.1. (**A**) Pb2; (**B**) Pd1; (**C**) Pd2; (**D**) Pd3; (**E**) Pj1; (**F**) Pj2; (**G**) Sd2; (**H**) chloroquine. Dotted lines indicate 50% viral inhibition. Data are expressed as the mean ± SD of three independent experiments (*n* = 3).

**Figure 3:**
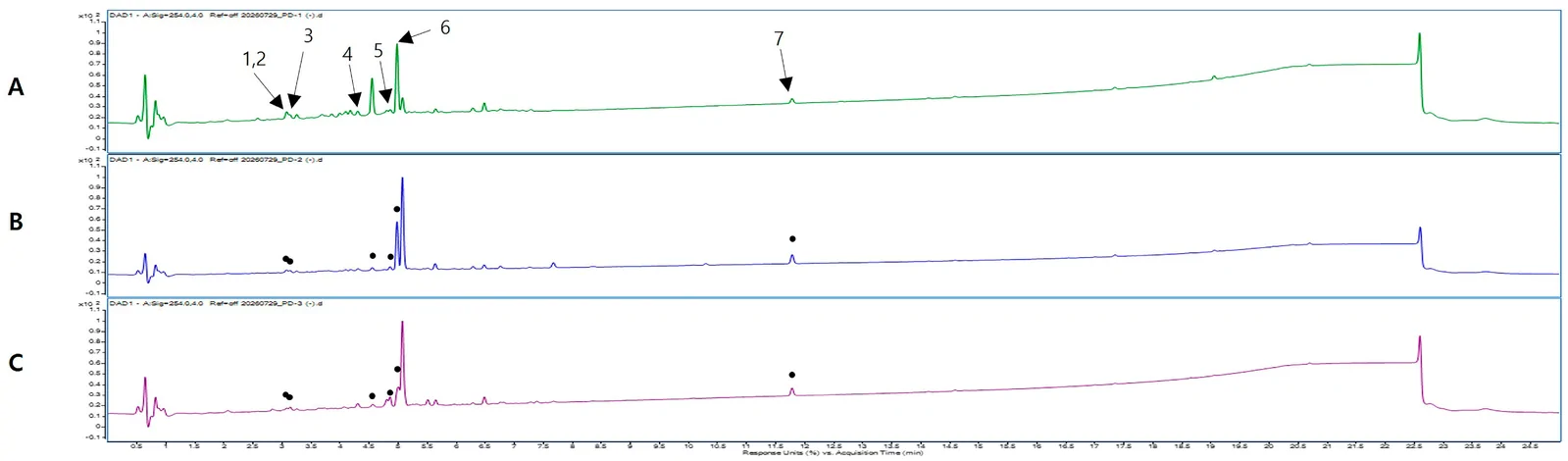
Representative UHPLC-PDA chromatograms (254 nm) of three independent *Peucedanum terebinthaceum* var. *deltoideum* extracts [(**A**) Pd1; (**B**) Pd2; (**C**) Pd3], showing the reproducibility of the characteristic chromatographic profile. Peaks 1–7 correspond to the characteristic constituents annotated based on UHPLC-Q-TOF-MS data using an in-house Personal Compound Database and Library (PCDL). The annotated compounds were identified as apiosylskimmin (1), chlorogenic acid (2), eleutheroside B (3), apterin (4), kaempferol 3-robinobioside (5), nicotiflorin (6), and decursin (7). Filled circles (•) in (**B**) and (**C**) indicate the retention times corresponding to the characteristic constituents identified in (**A**). The identities of compounds 1–7 are summarized in Table 4.

**Table 1:** Voucher specimens of examined taxa of *Peucedanum* and *Sillaphyton*.

Taxa (Extract ID)	Information on Voucher
*P. chujaense* (Pc)	South Korea, Jeollanam-do, Sinan-gun, Heuksan-myeon, 21 September 2021, J.-H. Song, *SJH_KIOM-2021-820* KIOM2021820 (extraction No. 3-24-0014)
*P. hakuunense* (Ph)	South Korea, Jeollanam-do, Gwangyang-si, Ongryong-myeon, Baegunsan Mountain, 12 August 2021, J.-H. Song & S. Yang, *SJH_KIOM-2021-569* KIOM 20210436 (extraction No. 3-24-0001)
*P. japonicum* (Pj)	South Korea, Jeju-do, Jeju-si, Yongdamsam-dong, 10 October 2020, J.-H. Song & S. Yang, *YSG_KIOM-2020-256* KIOM 20210452 (extraction No. 3-24-0012)
*P. miroense* (Pm)	South Korea, Gangwon-do, Samcheok-si, Miro-myeon, Naemiro-ri 7 September 2021, J.-H. Song & S. Yang, *YSG_KIOM-2021-646* KIOM 202101023797 (extraction No. 3-24-0006)
*P. terebinthaceum* var. *terebinthaceum* (Pb)	South Korea, Chungcheongbuk-do, Goesan-gun, Yeonpung-myeon, 5 August 2021, J.-H. Song, *SJH_KIOM-2021-567* KIOM 20210429 (extraction No. 3-24-0008)
*P. terebinthaceum* var. *deltoideum* (Pd)	South Korea, Gangwon-do, Yanggu-gun, Dong-myeon, 13 August 2021, J.-H. Song, *SJH_KIOM-2021-644* KIOM 20210432 (extraction No. 3-24-0010)
*P. tongkangense* (Pt)	South Korea, Gangwon-do, Jeongseon-gun, Hwaam-myeon, Seokgok-ri, 7 September 2021, J.-H. Song & S. Yang, *SJH_KIOM-2021-776* KIOM 2021776 (extraction No. 3-24-0004)
*S. podagraria* (=*P. insolens*) (Sd)	South Korea, Gangwon-do, Samcheok-si, Miro-myeon, Sanggeono-ri 6 September 2021, J.-H. Song & S. Yang, *YSG_KIOM-2021-605* KIOM 202101023797 (extraction No. 3-24-0016)

**Table 2:** Morphological characteristics of the aerial parts of the examined *Peucedanum* and *Sillaphyton* taxa.

Taxa	Diam. of Stem (cm)	Lower Cauline Leaf Type	Shape of Ultimate Segment of Leaves	Color of Surfaces of Leaves (ad/ab)	No. of Rays per Compound Umbel	No. of Flowers per Umbel	Shape of Mericarp
*P. chujaense*	0.6–0.9	1- or 2-pinnate	elliptic or obovate	green/ pale green	16–18	20–24	ellipsoid
*P. hakuunense*	0.4–0.6	1- or 2-ternate	narrowly lanceolate	green/ pale green	10–20	15–20	narrowly ellipsoid
*P. japonicum*	0.5–1.2	1- or 2-ternate	rhombic–ovate, obovate, or elliptic	glaucous/ glaucous	18–30	20–36	narrowly ellipsoid
*P. miroense*	0.4–0.7	2-pinnate	narrowly oblong–lanceolate to linear	green/ green	12–16	16–23	oblong
*P. terebinthaceum* var. *terebinthaceum*	0.3–0.6	2-pinnate	narrowly oblong to lanceolate	green/ green	10–22	20–34	narrowly ellipsoid
*P. terebinthaceum* var. *deltoideum*	0.3–0.6	2-pinnate	ovate to oblong	green/ green	10–22	20–34	narrowly ellipsoid
*P. tongkangense*	0.6–1.1	3-pinnate	narrowly oblong–lanceolate to linear	green/ green	16–18	15–25	narrowly ellipsoid
*S. podagraria* (= *P. insolens*)	0.1–0.4	1- or 2-ternate	ovate or ovate–oblong	green/ pale green	8–14	12–18	ovoid to ellipsoid

**Table 3:** Summary of the cytotoxicity and antiviral activity of *Peucedanum* and *Sillaphyton* extracts against the influenza A/Puerto Rico/8/34 (H1N1) virus.

Extract ID	Taxa	CC_50_ (µg/mL)	IC_50_ (µg/mL)	SI	Antiviral Activity
1-1 (Ph1)	*P. hakuunense*	>100 ^1^	>100	—	Inactive
1-2 (Ph2)	*P. hakuunense*	>100	>100	>0.704	Inactive
1-3 (Ph3)	*P. hakuunense*	94.812 ± 3.364	99.388 ± 0.022	0.954	Inactive
2-1 (Pt1)	*P. tongkangense*	>100	>100	—	Inactive
2-2 (Pt2)	*P. tongkangense*	>100	46.580 ± 5.286	>2.146	Inactive
2-3 (Pt3)	*P. tongkangense*	81.706 ± 0.548	>100	—	Inactive
3-1 (Pm1)	*P. miroense*	>100	89.965 ± 2.264	>1.111	Inactive
3-2 (Pm2)	*P. miroense*	94.087 ± 0.682	>100	—	Inactive
3-3 (Pm3)	*P. miroense*	>100	>100	—	Inactive
4-1 (Pb1)	*P. terebinthaceum* var. *terebinthaceum*	>100	>100	—	Inactive
4-2 (Pb2)	*P. terebinthaceum* var. *terebinthaceum*	>100	46.583 ± 5.185	>2.147	Inactive
4-3 (Pb3)	*P. terebinthaceum* var. *terebinthaceum*	>100	51.728 ± 3.254	>1.933	Inactive
5-1 (Pd1)	*P. terebinthaceum* var. *deltoideum*	>100	6.424 ± 0.367	>15.566	Active
5-2 (Pd2)	*P. terebinthaceum* var. *deltoideum*	>100	9.636 ± 0.728	>10.377	Active
5-3 (Pd3)	*P. terebinthaceum* var. *deltoideum*	>100	12.186 ± 0.960	>8.210	Active
6-1 (Pj1)	*P. japonicum*	>100	29.911 ± 5.598	>3.343	Inactive
6-2 (Pj2)	*P. japonicum*	>100	15.520 ± 3.334	>6.443	Active
6-3 (Pj3)	*P. japonicum*	>100	>100	—	Inactive
7-1 (Pc1)	*P. chujaense*	>100	>100	—	Inactive
7-2 (Pc2)	*P. chujaense*	>100	>100	—	Inactive
7-3 (Pc3)	*P. chujaense*	>100	>100	—	Inactive
8-1 (Sd1)	*S. podagraria*	>100	20.130 ± 5.551	>4.967	Inactive
8-2 (Sd2)	*S. podagraria*	>100	20.193 ± 3.948	>4.952	Inactive
8-3 (Sd3)	*S. podagraria*	>100	>100	—	Inactive
Pasa	*Peucedanum* sp.	>100	>100	—	Inactive
Paer	*Peucedanum* sp.	>100	>100	—	Inactive
Chloroquine	Positive control	25.000 ± 1.012	11.262 ± 0.942	2.220	Active

^1^ IC_50_ and CC_50_ values are presented as the mean ± standard deviation (SD). For values exceeding the maximum tested concentration (>100 µg/mL), SD values are not reported because 50% inhibition or cytotoxicity was not reached within the tested concentration range.

**Table 4:** Annotation of the characteristic constituents detected in *Peucedanum terebinthaceum* var. *deltoideum* (Pd) by UHPLC-QTOF-MS using an in-house Personal Compound Database and Library (PCDL).

Peaks	Compound Name	RT	Negative		Positive		Molecular Formula	Exact Mass
			*m*/*z*	Adduct	*m*/*z*	Adduct		
1	Apiosylskimmin	3.04	501.1236	[M+COOH]^−^	479.1159	[M+Na]^+^	C_20_H_24_O_12_	456.13
2	Chlorogenic acid	3.09	353.0870	[M−H]^−^	355.1021	[M+H]^+^	C_16_H_18_O_9_	354.10
3	Eleutheroside B	3.15	417.1391	[M+COOH]^−^	395.1310	[M+Na]^+^	C_17_H_24_O_9_	372.14
4	Apterin	4.32	469.1343	[M+COOH]^−^	447.1263	[M+Na]^+^	C_20_H_24_O_10_	424.14
5	Kaempferol 3-robinobioside	4.82	593.1500	[M−H]^−^	595.1656	[M+H]^+^	C_27_H_30_O_15_	594.16
6	Nicotiflorin	5.01	593.1505	[M−H]^−^	595.1662	[M+H]^+^	C_27_H_30_O_15_	594.16
7	Decursin	11.91	-	-	329.1387	[M+H]^+^	C_19_H_20_O_5_	328.13

## Data Availability

The publicly available plant-extracted dataset analyzed in this study was obtained from the Personal Compound Database and Library (PCDL).

## Abbreviations

The following abbreviations are used in this manuscript:

s. l.	Sensu lato
CPE	Cytopathic effect
MOI	Multiplicity of infection
PCDL	Personal Compound Database and Library
SI	Selectivity Index
IC_50_	Median inhibitory concentration
CC_50_	Median cytotoxic concentration
EC_50_	Median effective concentration
QTOF-MS	Quadrupole time-of-flight mass spectrometer
